# The American Public Health Association

**Published:** 1882-01

**Authors:** 


THE AMERICAN PUBLIC HEALTH ASSOCIATION.—The ninth annual meeting of this association was held at Savannah, Ga., beginning on November 29, and ending on December 2, 1881.

The meeting was well attended by members from all sections of the country. The proceedings were interesting and a considerable amount of good work was done. As usual, however, some of the papers read hardly rose to the level of mediocrity.

The address of the President, Dr. C. B. White, of New Orleans, was principally devoted to the question, whether there really is such a thing as sanitary science. Some of the defects in our present knowledge were tersely pointed out by the speaker. He referred to the apparent contradictions existing in the sanitary history of members of the proletariat, who, living among filth and other unwholesome surroundings, yet remained free from disease. These facts stand out in striking contrast to the assertions constantly made that cleanliness is not only "next to Godliness," but that it is indispensable to the preservation of bodily health.

As yet we have only seen a very meagre abstract of Dr. White's address. When it is published in full we shall, perhaps, make some more extended comments.

The following officers were elected for the ensuing year: Prof. R. C. Kedzie, of Michigan, President; Drs. E. M. Hunt, of New Jersey, and A. L. Gihon, U. S. Navy, Vice Presidents, and Dr. J. Berrien Lindsley, Tenn., Treasurer.

